# Serrated Lesions of the Colon-Rectum: A Focus on New Diagnostic Tools and Current Management

**DOI:** 10.1155/2019/9179718

**Published:** 2019-01-10

**Authors:** Gianluca Cassese, Alfonso Amendola, Francesco Maione, Mariano Cesare Giglio, Gianluca Pagano, Marco Milone, Giovanni Aprea, Gaetano Luglio, Giovanni Domenico De Palma

**Affiliations:** ^1^University of Naples “Federico II”, Department of Clinical Medicine and Surgery, Italy; ^2^Center of Excellence for Technological Innovation in Surgery, University of Naples “Federico II”, Italy

## Abstract

Prompt diagnosis and correct management of the so called “serrated lesions” (SLs) of the colon-rectum are generally considered of crucial importance in the past years, mainly due to their histological heterogeneity and peculiar clinical and molecular patterns; sometimes, they are missed at conventional endoscopy and are possibly implicated in the genesis of interval cancers. The aim of this review is to focus on the diagnostic challenges of serrated lesions, underlying the role of both conventional endoscopy and novel technologies. We will show how an accurate and precise diagnosis should immediately prompt the most appropriate therapy other than defining a proper follow-up program. It will be emphasized how novel endoscopic techniques may provide better visualization of mucosal microsurface structures other than enhancing the microvascular architecture, in order to better define and characterize specific patterns of mucosal lesions of the gastrointestinal tract. Standard therapy of SLs of the colon-rectum is still very debated, also due to the relatively lack of studies focusing on treatment issues. The high risk of incomplete resection, together with the high rate of postcolonoscopy interval cancers, suggests the need of an extra care when facing this kind of lesions. Given this background, we will outline useful technical tips and tricks in the resection of SLs, taking aspects such as the size and location of the lesions, as well as novel available techniques and technologies, other than future perspectives, including confocal laser endomicroscopy into consideration. Follow-up of SLs is another hot topic, also considering that their clinical impact has been misunderstood for a long time. The incidence of the so called interval colorectal cancer underlines how some weaknesses exist in current screening and follow-up programs. Considering the lack of wide consensus for the management of some SLs, we will try to summarize and clarify the best strategies for their optimal management.

## 1. Introduction

Colorectal carcinoma (CRC) is the most common gastrointestinal malignancy as well as the second cause of cancer-related death in USA [1], accounting for almost 50000 deaths and 130000 newly diagnosed cases. In addition, it is responsible for a high disability rate.

Studies in the field of “cancer biology” and prevention led to the concept that colorectal cancer is actually a heterogeneous disease, developing from different types of “precursors” and, at least, through three different molecular patterns, as further discussed below.

Both clinical and preclinical researches focused for decades on the role of “adenomatous polyps” as cancer precursors; the adenoma-carcinoma sequence, involving the accumulation of different progressive genetic mutations (APC, K-RAS, DCC, and p53), is widely recognized, and around 50-70% of CRC arises from conventional adenomas and is “microsatellite stable” [2].

Nevertheless, despite the fact that the malignant potential of adenomatous polyps is undeniable, more recent studies emphasized how around 10-30% of CRCs develop through a different pattern: from a genetic point of view, these cancers show high levels of microsatellite instability and/or BRAF mutations. From a clinical and histological perspective, these latter cancers arise from the so-called serrated lesions [3, 4].

As far as the histological aspect is concerned, SLs are characterized by a “serrated” (or saw-toothed) appearance of the epithelial glandular crypts.

Traditionally, all colonic polyps showing these histological features were referred to as “hyperplastic polyps” (HPs) and supposed not to have any malignant potential [5].

Afterwards, in the late 80's, some reports described the association between hyperplastic lesions and CRC [6, 7]. Finally, serrated lesions showing a neoplastic progression were referred to as “serrated adenomas” [8] (SAs).

SAs are also involved in the so-called “interval cancer” [9]. This kind of cancer is caused by a rapidly progressive precursor lesion, often difficult to detect; more, the possibility of an incomplete endoscopic resection is another risk factor. Sessile serrated adenoma/polyps with cytologic dysplasia (SSA/P-Ds) meet these criteria, impairing the role of screening colonoscopies in CRC prevention [10]. They usually have a right-side colonic location, show molecular hypermethylation, and may appear relatively soon after a complete colonoscopy [11, 12]. All these data support the hypothesis that around 10-30% of CRCs arise from nonadenomatous precursors, with a different genetic pattern including microsatellite instability, perhaps involving BRAF mutations and nuclear hypermethylation [13].

On the other hand, SLs represent a diagnostic challenge too, because they are often subtle, flat lesions, with indistinct margins, and covered by adherent mucous. Neoplasms arising from serrated lesions are often located in the proximal colon, and the difficulty to endoscopically detect such lesions might account for the decreased role of colonoscopy in protecting against right colon cancers.

The present review aims to provide a literature overview, emphasizing how novel available endoscopic technologies are helpful in dealing with the diagnosis and treatment of SLs of the colon-rectum. Next frontiers have been also discussed, particularly the role of confocal laser endomicroscopy.

## 2. Classification and Histology

According to the World Health Organization (WHO) [14], serrated lesions are currently classified into three main categories as follows: (1) hyperplastic polyps (HPs), (2) sessile serrated adenoma/polyps (with or without dysplasia) (SSA/Ps), and (3) traditional serrated adenomas (TSAs). This classification is mainly based on their histological features. In the WHO classification, at least three crypts (or two adjacent crypts) must have these features for the diagnosis, while according to the American Gastroenterological Association, only one crypt showing them is sufficient for the diagnosis of SSA/Ps [15]. This discrepancy can induce a significant impact on SSA/P prevalence.

A short histological background is of crucial importance in order to understand the issues regarding the diagnosis and proper management of SLs.

Until the late nineties, only one type of colorectal serrated polyp, the hyperplastic polyp, had been studied and recognized, which was not considered a precancerous lesion. This concept has then been changed thanks to Torlakovic and Snover who examined and first described six patients with numerous serrated polyps and also with colonic adenocarcinoma or numerous adenomatous foci [16]. The authors gave the name *serrated adenomatous polyposis* to the condition observed. Some pathologists preferred the name *sessile serrated polyp* rather than *sessile serrated adenoma*, because of the absence of typical adenoma-like dysplasia [17]. Nowadays, the terms “sessile serrated adenoma/polyp,” “sessile serrated polyp,” “sessile serrated adenoma,” or “sessile serrated lesion” essentially represent the same lesion. The need to clarify this situation led the WHO to name these entities as “sessile serrated adenoma/polyps,” abbreviated as SSA/Ps. Regardless of the definition, the key thing to take into account is the understanding of the existence of colorectal cancers associated with serrated lesions. Nevertheless, important issues still exist regarding the diagnosis of these lesions, mainly due to a great interindividual variability among pathologists in the correct interpretation and description of SSA/Ps [18].

Further histopathology details of serrated lesions of the colon-rectum are discussed below.

Hyperplastic polyps (HPs) are characterized by straight serrated crypts, extending from the polyp surface to the muscularis mucosae, without distortion; no horizontal or irregular branching is generally observed, with the “serration” being more frequent in the upper half of the polyp. Based on cytological features, the three main types of hyperplastic polyps have been described: (1) microvescicular HP (MVHP), (2) goblet cell HP (GCHP), and (3) mucin-poor HP; this differentiation mainly depends upon cell characteristics of the lining epithelium.

Sessile serrated adenoma/polyps (SSA/Ps) were previously referred to as “large” or “giant” HPs [19, 20]. They are mainly located in the right colon, accounting for 3-9% of all colorectal polyps [14, 21]. The peculiar feature of these types of lesions lies in their growth pattern within the serrated glands: they show crypt dilation, irregular branching crypts, and horizontally arranged crypts, with a sort of lateral growth pattern (T-shaped/L-shaped or boot-like crypts). These characteristics are mainly found at the basal portion of the crypt. Another feature of these polyps is that they often produce a big amount of mucin that often fills in the crypt lumen producing dilation, other than covering the surface of the polyp. The crypt may also “herniate” through the muscularis mucosae, giving a “pseudoinvasive” pattern. A crucial histological subcategory of SSA/Ps depends on the presence of *cytological dysplasia* (SSA/P-Ds). SSA/P-Ds present a portion of the polyp with the typical serrated pattern and another with dysplastic areas, with hyperchromatic, pseudostratified nuclei and increased mitoses, with a nuclear atypia. Different types of dysplasia are described by WHO: conventional adenomatous dysplasia and serrated dysplasia. The *conventional adenomatous-type dysplasia* may range from a low grade to a high grade, although it is usually recommended to treat these polyps always as “advanced polyps,” no matter the grade of dysplasia identified [15]. Another type of dysplasia, termed *serrated dysplasia*, can also develop in SSA/Ps. It is characterized by cells with a more cuboidal shape and eosinophilic cytoplasm, enlarged vesicular nuclei, and prominent nucleoli. Serrated dysplasia in SSA/Ps is frequently characterized by a complex pattern that corresponds to variably lower levels of expression of MLH1 by immunohistochemistry, until a complete loss, suggesting that transition to dysplasia is associated with methylation-induced silencing of tumor-suppressor genes, one of which is MLH1 [22, 23].

A recent Australian study described four subgroups of SSA/Ps with dysplasia, trying to correlate different dysplasia patterns with a specific molecular pathway. An association between “*minimal deviation dysplasia*” and the loss of MLH1 expression was suggested. On the other hand, “*serrated dysplasia*” and “*adenomatous dysplasia*” patterns were also described, keeping a normal MLH1 expression; the last pattern was defined as “*not otherwise specified dysplasia*” [24].

Traditional serrated adenomas (TSAs) are usually found in the left-sided colon with a “protruding” morphology [25]. As for the histology, they present a distorted tubulovillous or villous configuration, characterized by multiple, ectopic, proliferating crypts. Both serrated and conventional adenoma-like dysplasias can be found in TSA: it has been reported that up to 25% of TSA can present cytological dysplasia, with “intramucosal adenocarcinoma/high-grade dysplasia” found in 8% of them [26].

## 3. Molecular Pathways

As previously mentioned, the transforming pathway from serrated adenoma to cancer seems to be different from the traditional adenoma-carcinoma sequence followed by tubular or tubule-villous adenoma, in which biallelic inactivation of the *APC gene* is an initiating event. Around 15% of colorectal cancers show high-level “microsatellite instability” (MSI), other than methylation of nuclear CpG islands: this pattern is referred to as the CpG methylator phenotype (CIMP) [27]. The mechanism of action of methylation is based on the epigenetic silencing of a large number of tumor-suppressor genes, including MLH1, that creates a dysfunction of DNA mismatch repair and subsequent MSI, due to the alteration of normal mismatch repair, the same defective condition present in the Lynch syndrome. Sporadic colorectal cancers showing MSI also have CIMP, and it is gaining increasing acceptance that they probably evolve from serrated adenoma/polyps rather than from conventional adenomas [28–30]. It has been reported that a high proportion (up to 92%) of SSA and TSA show CIMP, thus emphasizing that they represent the precursor of sporadic MSI-H cancers [31].

Another genetic clue in the SSA/P pathway must be identified in BRAF mutation. It is well known that around 30% of colorectal carcinomas or large adenomas present an activating mutation of the K-RAS oncogene [32, 33]; on the other hand, around 10% of CRCs harbour an activating mutation of the BRAF oncogene (V600E) [32]; mutations in either K-RAS or BRAF are common in colorectal tumors but are mutually exclusive, because they encode kinases that belong to the mitogen-activated protein kinase (MAPK) cascade. It has been reported that SSAs carry a high frequency of activating BRAF mutations (75-82%) [34], which also seems to be a marker of MSI-H cancers. All these data seem to demonstrate that the malignant transformation of SSA occurs through a novel pathway involving MSI and BRAF mutations [35]. Spring et al. [13] report a series of 190 unselected consecutive patients; polyps were detected in 72% of patients. Most of them (60%) were traditional adenomas, followed by hyperplastic polyps (29%), SSA/Ps (9%), and TSAs (0.9%). The authors also searched for BRAF mutations, whose detection rate was very high in SSA/Ps (78%), TSAs (66%), and microvescicular hyperplastic polyps (70%), whereas K-RAS mutations were only identified in 8% of SSA/Ps. It is interesting also that microvescicular hyperplastic polyps can have these molecular features, leading Yang et al. to suggest that SSA/Ps may have evolved from this type of lesions [36]. On the other hand, BRAF mutation was identified in only 1 patient with tubular adenoma, emphasizing that there is likely a link between BRAF mutations and serrated lesions.

SSA/Ps with cytological dysplasia also present a greater molecular heterogeneity as compared with other proximal colonic lesions, expressing not only the typical BRAF mutations and CIMP but also p16 and MLH1 methylations, leading to microsatellite instability.

Finally, Gala et al. showed that germline mutations in some genes involved in senescence pathways (*ATM*, *PIF1*, *TELO2*, *XAF1*, and *RBL1*) were associated with the development of multiple SSAs (odds ratio [OR] = 3.0; 95% confidence interval). In particular, nonsense mutations in *RNF43* were also associated with multiple serrated polyps (OR = 460; 95% confidence Interval), appearing as a regulator of ATM–ATR DNA damage response [37].

## 4. Endoscopic Features of Serrated Lesions of the Colon-Rectum

The role of colonoscopy is certainly crucial in proper diagnosis and management of SLs and also in the light of the terrific technological innovations, which allow an effective and reliable differential diagnosis of SLs.

### 4.1. Hyperplastic Polyps (HPs)

**Figure 1:**
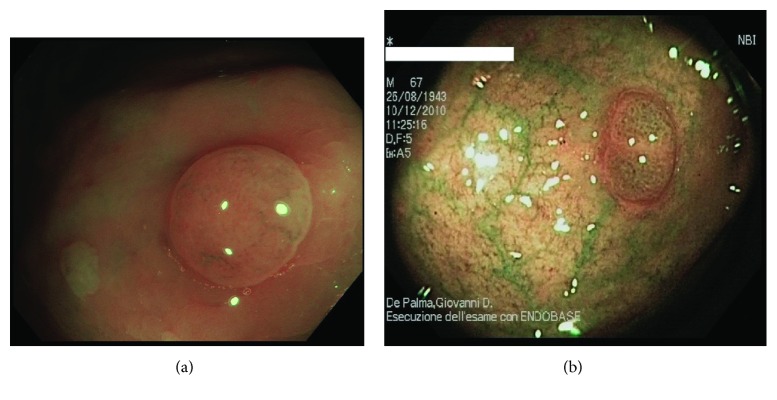
Endoscopic view of a hyperplastic polyp. (a) White-light endoscopic view showing a pearl-colored pale lesion of the sigmoid colon, about 0.5 cm diameter, Paris classification 0-Is. (b) NBI (narrow-band imaging) view of a hyperplastic polyp of the sigmoid colon, about 0.5 cm diameter, Paris classification 0-Is, prevalent type II (stellate) pits.

Figure 1 shows the endoscopic features of HPs: they are generally smooth, protruding and pale, and darker in the border areas with the surrounding healthy mucosa. They are small-sized (less than 5 mm) and found more frequently in the left colon and rectum. They also tend to be flat or even depressed at insufflation. HPs characteristically present with primarily asteroid-shaped pits (type II pits) at magnifying endoscopy (ME) [38].

### 4.2. Sessile Serrated Adenoma/Polyps (SSA/Ps)

Similar to HPs, SSA/Ps show a pale colour at endoscopy. They are usually flat or sessile, with indistinct borders. In general, these lesions are larger than 10 mm and more likely localized in the right-sided colon. More, they can present a characteristic yellowish mucous cap. When crystal violet staining is performed, the orifices are magnified and are clearly seen with their wide opening and are referred to as II-open pit [39]. Due to these features, differentiation between SSA/Ps and HPs can be difficult, also considering that these lesions can share some histological features.

Considering the potential for malignancy arising from SSA/Ps, it is necessary to shed a light on the endoscopic features of SSA/Ps with and without dysplasia.

A recent study by Muramaki et al. can provide excellent insights, in order to guide endoscopists to discriminate between SSA/Ps with and without dysplasia [40, 41]. Four endoscopic aspects were identified as worthy of suspicion: pedunculated or semipedunculated morphology, double elevations (63.4% of SSA/Ps with cytological dysplasia vs. 4.6% without dysplasia), central depressions, and marked reddishness (39% in those with dysplasia, 3.4% in those with no dysplasia, and 85.7% when an invasive carcinoma is present). The presence of at least one of these four features was found to be highly sensitive (91.7%) in predicting dysplasia/carcinoma within SSA/Ps, with a specificity of 85.3% (95% confidence interval). In addition, magnifying chromoendoscopy revealed that a variety of type III_L_, IV, V_I_, and V_N_ pit patterns were present in most of the SSA/Ps with dysplasia or carcinoma.

Sano et al. recently highlighted other morphological aspects related to SSA/Ps with cytological dysplasia. In particular, the presence of large/small nodules on the surface of the polyps and a partial protruding morphology of the lesions are strongly predictive of dysplasia (sensitivity, 46.2%; specificity, 97.3%; positive predictive value, 60%; and negative predictive value, 95.4%) [42].

**Figure 2:**
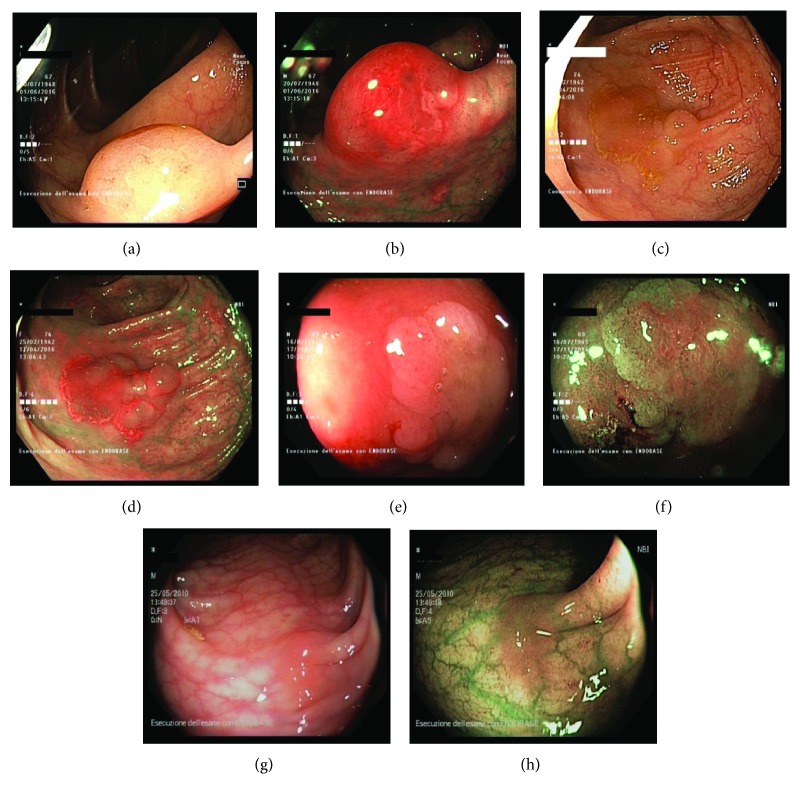
Endoscopic pictures showing the main morphologic characteristics of sessile serrated adenoma/polyps (SSA/Ps). All lesions are located proximally to the splenic flexure. (a) Typical sessile lesion with mucous coat, dome shape, and indistinct edges. (b) The mucus looks red under NBI (narrow-band imaging). (c) SSP at the hepatic flexure encircled by a rim of debris and obscuring the course of the submucosal vein. (d) The same lesion seen in 2c at NBI. (e) Type O-IIb lesion of the ascending colon, which displays a cloud-like surface. (f) The same lesion seen in 2e at NBI. (g) Flat SSP in the distal transverse colon identified only by a subtle nodular appearance of the surface mucosa. (h) The same lesion seen in 2g at NBI (narrow-band imaging).

Some of peculiar endoscopic characteristics of SSA/Ps are shown in Figure 2.

### 4.3. Traditional Serrated Adenomas (TSAs)

TSAs usually appear as protruding lesions, even if they may be flat in some cases. They are generally bright red, villous lesions, often associated with a type II pit pattern at the base. The macroscopic gross type is reported to be “pine cone-shaped” or “coral-shaped” via conventional observation. Magnifying endoscopic findings also reveal that the type IV pit pattern is often present, making the discrimination from traditional adenomas easy.

On the other hand, endoscopy sometimes hardly discriminates between TSAs and SSA/Ps, due to their similar pit pattern profile. Some endoscopists have used the terms type III H and IV H pits or type IV to serrated pit pattern to differentiate from conventional villous adenomas [43].

Despite the potential of modern endoscopic technologies, reports from literature show a wide variety in the detection rate of SLs during endoscopy. Hetzel et al. [21], for example, in a retrospective study performed on patients undergoing colonoscopy from 2006 to 2008 at the Boston Medical Center, report different detection rates among endoscopists even within the same center; specifically, 4355 polyps from 7192 colonoscopies were analyzed and variability was observed for each type of lesions. Adenoma detection ranged from 13.5 to 36.4 patients per 100 colonoscopies (7.9-26.1 for proximal adenomas), HP detection ranged from 7.7 to 31.0 patients per 100 colonoscopies (1.1-6.7 for proximal HPs), and SSA/P detection ranged from 0.0 to 2.2 patients for 100 colonoscopies (0.0-1.4 for proximal SSA/Ps). These variabilities of polyp detection among endoscopists were higher than predicted from the random error alone in adenomas, HPs, and SSAs (*P* < 0.001, *P* < 0.001, and *P* = 0.020, respectively). The detection rate of dysplastic serrated polyps (DSPs) and adenocarcinomas did not vary significantly between endoscopists (*P* = 0.823 and *P* = 0.391, respectively).

## 5. Role of Image-Enhanced Endoscopy (IEE) in the Diagnosis of Serrated Lesions

New endoscopic technologies allow both a better anatomical definition and an earlier recognition of lesions, potentially improving patients' prognosis by providing better visualization of mucosal microsurface structure and microvascular architecture.

*Endoscopic autofluorescence imaging* (*AFI*) produces real-time pseudocoloured images based on the detection of natural tissue fluorescence generated from endogenous fluorophores (collagen, nicotinamide, adenine dinucleotide, flavin, and porphyrins) through the induced emission by excitation light.

*Narrow-band imaging* (*NBI*) is an optical image technology that enhances structural mucosal patterns (pit patterns), as well as mucosal/submucosal vessels; a special electronic filter is activated to use specific blue and green wavelengths, thus enhancing details and vascularization of the mucosal surface [44].

Novel technologies are demonstrated to be able to improve the detection rate of these subtle lesions; as previously mentioned, Spring et al. [13] reported that 9% of all the lesions identified were SSA/Ps. In this study, the authors reported a 9% prevalence of SSA/Ps, 60% for conventional adenomas, 29% for HPs, and 0.7% for TSAs. As previously stated, SSA/Ps are more frequently located in the ascending colon (75%) and associated with burden polyps, BRAF mutations, and the female sex. This quite high detection rate was very likely facilitated by the implementation of magnification chromoendoscopy; previous reports, in fact, have estimated a prevalence of only 2% [45].

Saito et al. [43] describe the most important features of serrated lesions found at image-enhanced endoscopy (IEE), including autofluorescence imaging (AFI), narrow-band imaging (NBI), and infrared imaging. Below, we list the most important features of HPs, SSAs, and TSAs.

### 5.1. Hyperplastic Polyps (HPs)

HPs are visualized as dark-green coloured at AFI, similar to the normal surrounding mucosa.

On NBI, we do not observe dilation of the capillary vessels surrounding glands but a type II pit pattern is usually present. At IEE, as in conventional endoscopy, the HPs appear to be similar to the normal colon mucosa.

### 5.2. Sessile Serrated Adenomas (SSA/Polyps)

At AFI, SSA/Ps are visualized as dark-green coloured, similar to the HPs, despite the fact that this technique as a standard diagnostic method still needs to be validated.

Endoscopic discrimination of SSA/Ps with and without cytological dysplasia is usually really hard to obtain; in fact, the typical colour changing to magenta is possible to visualize in both SSA/Ps with and without dysplasia, other than in HPs.

In a pilot study, Boparai et al. [46] reported that both AFI and NBI are usually not reliable to discriminate between HPs and SSA/Ps, even using the pit pattern profile or the so called “vascular pattern intensity” (VPI). On the other hand, these features are effective in differentiating HPs from conventional adenomas. Nakao et al. [47] showed that the presence of a mucous cap together with dilated crypts might be helpful in the differentiation of SSA/Ps from HPs. They retrospectively examined 25 HPs and 46 SSPs and investigated with autofluorescence imaging (AFI) and magnifying endoscopy with narrow-band imaging (ME-NBI). The evaluation was focused on colour changes, capillary dilatation, and the existence of a mucous layer on the surface of the tumor. They concluded that finding a mucous cap or dilated pits (II-d pit) could be helpful in differentiating SSA/Ps from HPs with a good level of diagnostic accuracy.

### 5.3. Traditional Serrated Adenomas (TSAs)

TSAs typically appear of magenta colour when observed at AFI. Protruding TSAs of villous type usually show an intermediate colour in between magenta and dark green, while superficial TSAs are usually magenta (the colour may also vary depending on the grade of dysplasia).

At NBI, protruded-type TSAs usually show gland orifices in whitish colour, with the interstitial capillaries in blackish-brown colour; on the other hand, superficial TSAs do not present vessel dilation (different from protruding lesions). A blackish crypt dotted orifice is also visible within the lesions; as this feature can be found in SSA/Ps as well, the discrimination between these entities can be difficult at NBI [43].

## 6. Management of Serrated Lesions

### 6.1. Major Concerns about Serrated Lesions' Management

No clear consensus exists regarding the appropriate treatment and surveillance strategy for SLs of the colon-rectum. In a multicenter prospective study, involving 13 institutions and a total of 4000 snare polypectomies, Heldwein and colleagues suggest that the right colon location and the size of the lesions should lead to a careful evaluation regarding the risk/benefit implications of endoscopic removal [48]. It is generally recommended to remove all the SLs, with the exception of small (<5 mm) serrated-appearing lesions of the rectosigmoid colon that, when multiple, can be randomly sampled for histology [15]. Endoscopic resection principles remain the same compared to those for conventional adenomas; certainly, sometimes, removal of SLs can be challenging, in the case of large, flat lesion, if margins are not completely clear; such margins become even harder to identify after submucosal injection [49]. These technical aspects can be responsible for the higher rate of incomplete resection of SSA/Ps compared with conventional adenomas, as it was demonstrated in the CARE study, a double-center prospective study, that analyzed 1427 patients. In this paper, the incomplete resection rate resulted to be 31% for SLs (which were only 42) against 7% for nonserrated ones, with a peak of 48% for polyps larger than 10 mm [50]. These higher rates of incomplete resections can at least partially explain the association with the interval postcolonoscopy CRCs.

### 6.2. Resection Techniques

Resection techniques and indications may vary among centers and sometimes also depend on endoscopists' experience and preferences. Our approach, as a referral center for colorectal diseases, is based on tailored procedures taking the size, the location, and the type of lesions into consideration and also based on available evidence.

For smaller SLs (<10 mm) located at the right colon, standard polypectomy techniques can be used; in particular, cold snaring [51] has proved to be safe and effective [52] and so does electrocautery snaring. The use of submucosal lifting can be helpful in the endoscopic management of flat lesions.

**Figure 3:**
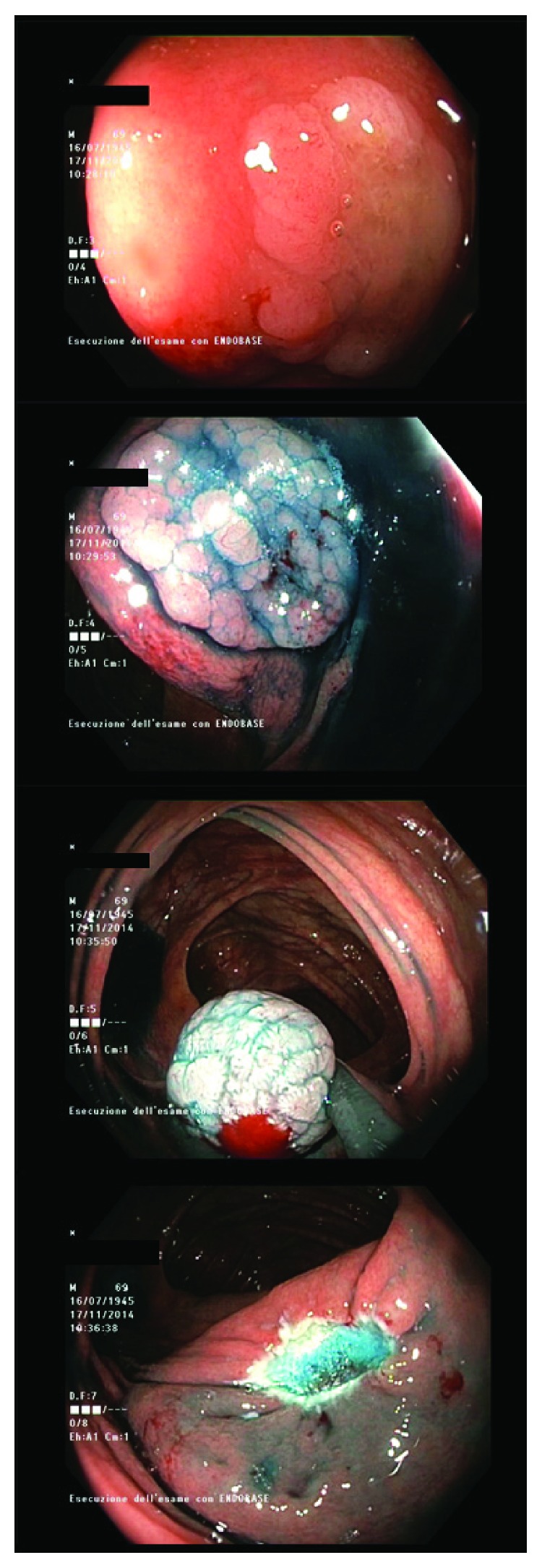
Different phases of the removal of a serrated adenoma of 1.5 cm: from the lifting of the flat lesion until the resection with an electrocautery snare.

For lesions larger than 10 mm, choosing the appropriate technique depends on both the lesion's features and the endoscopist's skills. According to our experience, we suggest to follow the current trends and recommendations for the management of large nonpolypoid colorectal polyps. Furthermore, the higher risk of incomplete resection for large SLs [50], together with the higher complications' rate associated with the removal of large sessile polyps in the right colon, suggests the possibility to introduce a threshold volume to improve the outcomes [53]. This should also probably encourage the treatment of large flat lesions of the right colon in referral centers with local expertise, as also recommended by the British Society of Gastroenterology [54]. A double-center retrospective study based on the analysis 251 SSA/Ps > 10 mm showed that a standard technique with submucosal saline injection and electrocautery snare is an optimal choice, with a good safety profile and an acceptable recurrence rate as low as 4% [55].  Figure 3 shows the main technical stages of such procedure.

Piecemeal resection is probably easier to perform for lesions larger than 20 mm; nevertheless, another colonoscopy is usually recommended 3-6 months after the first procedure, to make sure the excision was complete, bearing in mind a potential higher risk for recurrence [56]. With this regard, a recent prospective Australian study analyzing a total of 2000 lesions (323 SSA/Ps in 246 patients and 1527 adenomas in 1425 patients) showed that the risk of recurrence after piecemeal resection is lower for SSP > 20 mm than for adenomatous lesion, although not negligible (16% vs. 6.3% after 6 months, 20% vs. 7% after 12 months) [57]. This kind of resection has demonstrated to be safe and easy to perform, with 5.3% of complication rate [48]. On the other hand, considering more complex procedures like the endoscopic submucosal dissection, the potential benefit of a more radical resection needs to be counterbalanced by higher complication rates, with colonic perforation reported up to 6-7% [58, 59] and technical challenges [60]. Furthermore, the malignancy of these lesions is limited, perhaps not justifying these drawbacks.

Other recent and alternative snare techniques have been proposed for sessile lesion resections, but they cannot be routinely recommended, considering the current lack of evidence for a clear advantage. In particular, a prospective observational study suggests the effectiveness and the safety of underwater EMR [61], which uses intraluminal water in order to separate the mucosa and submucosa from more external layers; furthermore, this technique seems to be very easy to carry out, with no need of specific training [62]. Another method seems to be appropriate to deal with smaller flat lesions: the use of suction to make pseudopolyps easier to remove by a snare [63]. Recently, piecemeal cold snare polypectomy has also shown a high effectiveness with the important benefit of avoiding thermal injury [64, 65]. Lastly, surgical resection is rarely necessary but sometimes required in case of serrated lesions not amenable of endoscopic removal or in case of multiple lesions of the right colon.

### 6.3. Recommended Surveillance Program

Endoscopic surveillance is strongly recommended for patients with SLs. The role of SLs in the genesis of interval colon cancers suggests very close colonoscopy intervals [66, 67].

Recommendations on surveillance programs mainly reflect expert opinions to date.  Table 1 shows the most recent recommendations for surveillance programs of serrated class lesions according to the most important international experts' societies. We also favour the international consensus panel, which is detailed and conservative enough, taking the practice parameters from ASGE and ESGE into account [15, 68]. A five-year follow-up period is usually required for SSA/Ps without dysplasia and SSA/Ps that is 10 mm or smaller in size; on the other hand, a three-year interval follow-up is recommended for SSA/Ps with dysplasia and SSA/Ps larger than 10 mm; finally, a two-year interval follow-up is recommended for serrated polyposis.

It should be emphasized again that most of these recommendations are mainly based on expert opinions rather than on good-quality evidence. The major lack of consensus particularly concerns the unclassified SLs and the coexistence of SLs and conventional adenomas. Randomized and properly designed studies with wider populations are required, considering that even the British Society of Gastroenterology (BSG) and the United States Multi-Society Task Force (USMTF) admit a “weak and low-quality” evidence [51, 69].

## 7. Serrated Polyposis Syndrome

Serrated polyposis syndrome (SPS) has been recently redefined according to the clinical criteria stated by the WHO [70]. These criteria are listed in Table 2, and the presence of at least one of these is necessary in order to make the diagnosis. With that being said, SPS involves a very heterogeneous pot of patients with different phenotypes and genotypes.

SPS is a condition characterized by an increased incidence of CRC. The exact risk of CRC in SPS is actually unknown. In a multicenter retrospective study involving 77 patients with SPS, 35% of patients were found to have a CRC (28.5% at the first colonoscopy and 6.5% developed at follow-up) [71]. Similar results come from other smaller series (from 10 to 38 patients) [72–75], whereas the risk to find a colorectal cancer at the first endoscopy or at follow-up in patients with SPS ranged from 25% to 70%. Furthermore, first-degree relatives of SPS patients show an increased risk for CRC compared to the general population [76].

In the light of current evidence and our institutional experience, an annual colonoscopy is advisable in order to remove the SLs from the right colon, leaving behind only lesions smaller than 5 millimetres. The management strategy should be tailored on the basis of the polyp burden and histological findings. Surgery is indicated when a cancer is found [15] or when the endoscopic control of the lesions is technically difficult or risky. The surgical resection should include the removal of the colonic segment with cancer or larger polyps. Annual endoscopic surveillance of the residual colon and rectum is recommended, even if more studies are required to assess a proper and tailored strategy.

## 8. Future Perspectives and Role of Confocal Laser Endomicroscopy

The development of proper diagnostic tools is demonstrated to be crucial to improve the detection rate of SLs, reducing the rate of missed lesions and perhaps the incidence of interval cancers.

With regard to technical advances, it is worth mentioning the role of ENDOCUFF® (Arc Medical Design, Leeds, England), which, more than a future possibility, should be probably considered an actual tool to improve the adenoma detection rate. We have recently demonstrated in a randomized back-to-back trial at our institution [77] that this disposable device, made of a double row of flexible finger-like projection and applied at the tip of the colonoscope, is able to increase the detection of small adenomas (<5 mm), perhaps leading to a better definition of surveillance programs and potentially reducing the rate of interval cancer.

All these considerations underline the importance of keeping up with technology, and from this standpoint, *confocal laser endomicroscopy* (*CLE*) [78, 79] might represent the next frontier. CLE is quite a novel technology providing “in vivo” high-magnified images of microscopic details of the gastrointestinal mucosa, during conventional endoscopy; the role of targeted molecular probe associated with CLE has also been described and perhaps might represent a promising tool for early detection of colonic dysplasia [80, 81].

CLE promises the fascinating scenario to pursue the goal of an “in vivo” histology, developing technologies able to provide a real-time histological diagnosis, achieved during the endoscopic examination itself. With this regard, CLE is perhaps one of the most promising tools developed with this aim, allowing endoscopists to obtain 1000-fold magnified histological images of the gastrointestinal mucosa [82].

Despite the fact that the use of CLE has not been introduced into routine clinical practice yet, preliminary experiences are extremely promising in many fields of gastrointestinal endoscopy, from the characterization and management of Barrett's esophagus to the identification of malignant and premalignant lesions of the colon-rectum [80, 83, 84], thanks to its ability to detect cellular and subcellular structure of the colonic mucosa, other than assessing details of superficial vascular architecture [78].

More, the diagnostic potential of CLE can be further enhanced by the use of contrast agents (e.g., the fluorescein) that can also be linked to peptides which will selectively link to dysplastic cells or even to antibodies which will allow the visualization of inflamed areas, expressing specific cytokine patterns [85]. Thus, the horizons of CLE might potentially expand towards the early detection of preneoplastic lesions and also the assessment of the inflammatory activity in inflammatory bowel disease (IBD) [86].

Given this background and its capacity to detect early subtle lesions of the gastrointestinal tract, another intriguing application of endoscopic confocal laser endomicroscopy is represented by the detection of the SLs of the colon-rectum.

### 8.1. CLE: Technical Aspects

CLE is based on tissue illumination with a low-power laser with subsequent detection of the fluorescence of the light reflected from the tissue itself through a pinhole, in order to collect the light emitted by a single focal plane, to keep a perfect focus on that portion, eliminating any noise coming from planes above or below. In other words, the system uses the light emitted by an argon blue laser (488 nm) that passes through the pinhole and is focused on the focal plane of interest. This light will then be reflected and refocused in the detection system by the same lens; this mechanism also explains the term “confocal,” referring to the alignment of both illumination and collection systems in the same confocal plane [87, 88].

All the signals coming from the focused plane are thus detected and measured, in order to create a greyscale image of the tissue, then digitized and reconstructed, whereas the brightness of each pixel corresponds to the intensity of fluorescent light detected. Once a series of scanning planes have been created, the “optical sections” obtained will be overlapped by a software, allowing to reconstruct the overall image, also called “optical biopsy,” which will be noninvasive, real-time, and in vivo.

CLE can be based on both tissue reflectance of fluorescence [89]; devices based on tissue reflectance do not need any contrast agent, but they also generate low-quality images and therefore have limited clinical applications. On the other hand, CLE can be implemented using topical and/or intravenous fluorescent contrast agents, generating high-resolution images, similar to those of traditional histology [89]. Fluorescein is the most commonly used contrast agent, and it can be administered either topically or intravenously. Intravenous administration is most commonly used, and fluorescein has proved to be safe, just provoking a self-limiting yellowing of the skin, eyes, and urine [90]. It binds to serum albumin, but the free portion leaks through the capillary system, permeating tissues and contrasting the surface of the epithelium and the extracellular matrix, for about 30 minutes [91]; thus, epithelial cells, cellular infiltrates, enterocytes, vessels, and erythrocytes can be visualized.

### 8.2. Current Applications of Confocal Laser Endomicroscopy to Colorectal Diseases

Current application for CLE in gastrointestinal (GI) endoscopy includes Barrett's Esophagus, biliary strictures, diagnosis and follow-up of colonic and gastric lesions [92]. Other possible future applications of CLE might also include the diagnosis of breast diseases [93], celiac disease [94], and ampullary lesions [95]. One of the first fields of application of CLE was represented by Barrett's esophagus; the research in this field has greatly contributed to the standardization of confocal image interpretation.

The need to standardize indications, terminologies, and image interpretation led to the adoption of a schematic and objective classification: the 2009 Miami classification was then created [96]. This classification is based on consensus of pCLE users during a meeting in Miami in February 2009. With regard to colorectal diseases, the authors defined the criteria for pCLE classification of colorectal polyps. The *normal colonic mucosa* is characterized by hexagonal, round crypt structures that is of honeycomb appearance, dark goblet cells, and regular narrow vessels surrounding the crypts, covered by a homogeneous epithelium with “black hole” goblet cells in the subcellular matrix; *hyperplastic polyps* show crypts with slit or stellate openings (pits) and dark epithelial borders, bright nonthickened uniform epithelium, and dark goblet cells while the vessel architecture usually presents and increases in pericryptic capillary; adenomatous polyps, on the other hand, have irregular or villiform structures, a dark and irregularly thickened epithelium, with a decreased number of goblet cells and mucin depletion; *adenocarcinoma* appears with completely disorganized villiform or lack of structure, dark and irregularly thickened epithelium, and very dilated vessels. Inflammatory changes have also been characterized, and *colitis patterns* are represented by crypt fusion and distortion, bright epithelium, and dilated and prominent branching vessels [97].

Buchner et al. [98] compared the sensitivity and the specificity of CLE with respect to chromoendoscopy, in identifying colorectal polyps. They analyzed 119 polyps from 75 patients, demonstrating how CLE is more sensitive and specific, with histopathology as a standard reference. In particular, the sensitivity was found to be 91% and 77% for pCLE and chromoendoscopy, respectively (*P* < 0.010), and 88% vs. 76% (*P* = 0.037) for polyps larger than 10 mm. Given this data, it is easy to understand how this novel technique has the potential to avoid unnecessary polypectomies or biopsies, leading to real-time management decisions.

Another intriguing possibility is to bind the fluorescein with proteins having high-binding affinity for dysplastic colonocytes in order to enhance the visualization of dysplastic tissue at pCLE; the use of such fluorescent molecular probes associated with CLE has demonstrated to be more feasible in the setting of “sporadic” colonic dysplasia than in UC-associated dysplasia [80, 81].

We have also previously demonstrated the possibility to identify vascular patterns of tumor neoangiogenesis in CRC at pCLE [78]. In fact, different morphological vascular patterns can be enhanced at pCLE, discriminating between the normal and malignant mucosa, characterized by an increased vessel diameter with irregular shape, branching patterns, defective flux, and fluorescein leakage due to increased vessel permeability.

### 8.3. CLE and Serrated Lesions of the Colon-Rectum

Endoscopic detections of serrated lesions, as previously emphasized, may often represent a challenge, considering that they are sometimes subtle, diminutive, flat lesions and can be missed out, despite the implementation of several techniques of “augmented endoscopy.” More, considering that most of these lesions are represented by HPs with a negligible risk of malignant progression, the correct endoscopic management is also debated.

**Figure 4:**
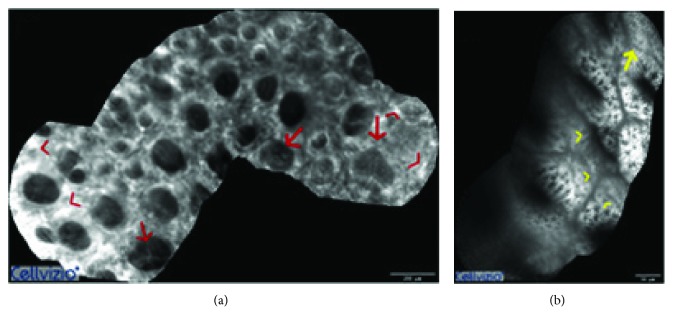
Confocal laser endomicroscopy of serrated lesions (from archives of Center of Excellence for Technological Innovation in Surgery). (a) Hyperplastic polyp, seen with low magnification: stellate crypt opening (red arrows) and dark epithelial borders. The vessel architecture demonstrates an increase in pericryptic capillary density (red arrowhead). (b) Sessile serrated adenomas/polyps (high magnification): branching crypts (yellow arrows) and an irregular architecture with abnormal-shaped crypts and dystrophic goblet cells (yellow arrowheads).

From this standpoint, the use of confocal laser endomicroscopy in the setting of colorectal SLs (Figure 4) is certainly appealing, due to its potential to improve the detection rate, facilitate targeted biopsies, and obtain a real-time histology, thus aiding for quick decision-making: resect or leave in place? En bloc or piecemeal resection? What about resection margins?

Unfortunately, these questions and the role of CLE in this field are still speculative, as there are very few studies comparing the pCLE with other endoscopic modalities.

Parikh et al. [99] led out one of the pivotal studies addressing the role of CLE in the diagnosis of SSA/Ps. This is the first report to describe the CLE features of SSA/Ps. The authors included seven consecutive patients with SSA/Ps, with a previous diagnosis at high-definition WL colonoscopy. 5 ml of 10% fluorescein sodium was administered intravenously to illuminate the extracellular matrix of the mucosal epithelium and lamina propria. Thus, they identified four main CLE features of SSA/Ps: (1) a mucus cap, presenting with a bright cloud-like appearance at CLE; (2) thin and branching crypts; (3) increased mucin, appearing with an increased number of goblet cells, as well as the presence of microvescicular mucin-containing cells; and (4) architectural disarray, appearing to have lack of regular circular crypts and the presence of dystrophic goblet cells. Compared with previously published reports about hyperplastic polyps [49], SSA/Ps have more mature goblet cells and mucinous cells within the bases of their crypts. Thanks to the CLE, the authors were able to visualize the mucus cap but not the orientation of crypts and they identified thin and branching crypts, without recognizing the base from the apex.

Despite the undeniable interest of this study, further research is necessary to further validate its findings. In particular, larger multicentric perspective studies are required to definitively assess the reproducibility of such criteria along with their predictive value in clinical practice. A prospective in vivo assessment of SSA/P versus non-SSA/P lesions, including hyperplastic polyps, would also perhaps be welcome.

Certainly, the widespread adoption of CLE in the everyday practice for detection of colorectal lesions is probably prevented by the low availability of this technology and its high cost. On the other hand, the learning curve seems to be quite easy and short. By the way, strongest evidence is probably required to definitively assess if CLE will deserve to become part of the diagnostic arsenal of the endoscopists, especially in the diagnosis of challenging entities like SLs [100].

## 9. Conclusion

This article provides an overview of the current knowledge and evidence regarding the diagnosis, management, and surveillance strategies of SLs of the colon-rectum and the serrated polyposis syndrome.

It is undeniable that the detection rate of SLs seems to be strongly correlated with the gastroenterologist's experience and skills. It has been recently demonstrated that an adequate diagnosis and training program for both gastroenterologists and pathologists are essential to implement the diagnostic performance [101], hopefully reducing the number of misdiagnosed lesions and the incidence of colorectal cancer. The importance of novel technologies has also been emphasized and, from this standpoint, as the “augmented endoscopy” is the present, confocal laser endomicroscopy might represent the next frontier.

## Figures and Tables

**Table 1:** Surveillance recommendations after complete removal of serrated polyps.

Histology	Size	Number	Localization	Surveillance (years)		
				International consensus panel	United States Multi-Society Task Force	British Society of Gastroenterology
HP	<10 mm	Any	Rectosigmoid	10	10	10
HP	≤5 mm	≤3	Proximal colon	10	N/A	10
HP	Any	≥4	Proximal colon	5	N/A	10
HP	>5 mm	≥1	Proximal colon	5	N/A	10
SSP/TSA	<10 mm	≤3	Any	5	5	10
SSP/TSA	>10 mm	1	Any	3	3	3
SSP/TSA	<10 mm	≤3	Any	3	5	10
SSP	>10 mm	≥2	Any	1-3	3	3
SSP w/ dysplasia	Any	Any	Any	1-3	3	3

HP: hyperplastic polyp; SSP: sessile serrated polyp; TSA: traditional serrated adenoma; SSP w/ dysplasia: sessile serrated polyp with cytological dysplasia; proximal colon: proximal to the sigmoid.

**Table 2:** WHO criteria for the diagnosis of SPS.

World Health Organization clinical criteria for the diagnosis of serrated polyposis (at least one criteria must be met)	
1	Five or more serrated polyps proximal to the sigmoid colon, two of which bigger than 10 mm in diameter
2	Any number of serrated polyps occurring proximal to the sigmoid colon in an individual who has a first-degree relative with serrated polyposis
3	More than 20 serrated polyps of any size distributed throughout the colon
